# Thyroid Volume in Systemic Sclerosis Patients: A Cross-Sectional Study

**DOI:** 10.7759/cureus.21422

**Published:** 2022-01-19

**Authors:** Suade Ozlem Badak, Bozkurt Gulek, Esra Kayacan Erdogan, Hulya Binokay, Eren Erken

**Affiliations:** 1 Rheumatology, Adana City Training and Research Hospital, Adana, TUR; 2 Radiology, Adana City Training and Research Hospital, Adana, TUR; 3 Rheumatology, Ankara City Training and Research Hospital, Ankara, TUR; 4 Biostatistics, Cukurova University Faculty of Medicine, Adana, TUR; 5 Rheumatology, Acıbadem Adana Hospital, Adana, TUR

**Keywords:** thyroid volume, thyroid ultrasonography, systemic sclerosis, modified rodnan skin score, modified medsger severity scale

## Abstract

Introduction: Systemic sclerosis (SSc) is a multisystemic disease. Thyroid involvement in systemic sclerosis is an overlooked issue. Our study aimed to evaluate the decreased thyroid volume in SSc. Also, we aimed to show the relationship between patients' thyroid volume and clinical and laboratory parameters.

Method: This was a single-center, cross-sectional study. Eighty-six patients were included in the study. A radiologist evaluated patients' thyroid volumes by ultrasonography. Demographic and clinical characteristics of the patients were recorded. Skin thickness was evaluated by the modified Rodnan skin score (mRSS) and the disease severity by the Medsger severity score (MSS). Findings were analyzed statistically.

Results: Thyroid volume was in the atrophic range in 53.5% of the patients. There was a significant negative correlation between thyroid volume and mRSS, MSS, and disease duration. Logistic regression analysis showed that mRSS and disease duration were risk factors for thyroid atrophy.

Conclusions: Many studies point out that thyroid autoantibodies are a cause of thyroid dysfunction in patients with SSc. However, in most of these studies, thyroid volume was not evaluated. As a result of our study, we saw that the major cause of thyroid dysfunction in our SSc patients was thyroid atrophy. Also, we observed that thyroid atrophy was more common in patients with interstitial lung disease. We would like to draw attention to the fact that thyroid dysfunction and volume changes increase with the disease's duration and severity in systemic sclerosis.

## Introduction

Systemic sclerosis (SSc) is a heterogeneous disease that may involve various organs and which may course with varying disease severity. The most prominent features of the disease are vascular abnormalities and fibrotic changes, accompanied by musculoskeletal, renal, pulmonary, cardiac, and gastrointestinal involvements. Thyroid affection in SSc is not as common as other organ involvements. But still it has been reported that thyroid dysfunction is encountered more frequently in SSc patients than in the general population [1]. Thyroid involvement frequently ends up with subclinical or clinical hypothyroidism, and may lead to conditions like cardiac dysfunction, atherosclerosis, myopathy, dysphagia, constipation, and certain psychological disorders. Thyroid dysfunction can aggravate SSc-related clinical symptomatology. Therefore, it is of utmost importance to not neglect the evaluation of the thyroid gland during the diagnosis and follow-up of SSc patients [2]. Lymphocytic infiltration, autoimmune catastrophe, and finally fibrosis of the thyroid gland, have all been blamed for the development of thyroid gland dysfunction due to SSc [1].

Numerous research studies have been conducted in order to investigate the relationship of SSc with autoimmune thyroid disease. But, on the other hand, only a few studies can be found in the literature which evaluate the thyroid volume and scrutinize the relationship between thyroid volume changes and SSc disease properties. The aim of this study was to evaluate the volume of the thyroid gland in SSc patients and demonstrate the relationship between thyroid volume and certain SSc disease characteristics, various clinical and laboratory parameters.

## Materials and methods

This was a single-center, cross-sectional study. Eighty-six (30 of the limited and 56 of the diffuse subtype) patients who had applied to the Rheumatology Department's outpatients' clinics and who had been diagnosed with diffuse or limited SSc, based on the American College of Rheumatology and European League Against Rheumatism (ACR and EULAR) 2013 criteria, were enrolled in the study [3]. Patients were classified as having diffuse or limited SSc, according to LeRoy's criteria [4]. All of the patients were from the same province. Patients with the following conditions were not included in the study: age under 18 years, overlap syndrome, a history of thyroidectomy, pregnancy, lactation, a history of neck irradiation, malignancy, I-thyroxine therapy, and intake of drugs that may affect thyroid functions. Data concerning age, gender, disease duration, and immunosuppressive therapy history were all recorded. The disease's duration was defined as the time between the onset of the first non-Raynaud's symptom and the study visit. Four patients had previously been treated with mycophenolate mofetil, while 36 patients had received prior cyclophosphamide therapy. All data concerning interstitial lung disease, pulmonary arterial hypertension (PAH), and gastrointestinal system (GIS) involvement were drawn from the hospital database. The diagnosis of interstitial lung disease (ILD) was made by respiratory function test, carbon monoxide diffusion test, high resolution computed tomography, and pulmonary arterial hypertension (PAH) was diagnosed by echo and right heart catheterization. Gastrointestinal system involvement was determined based on symptoms and gastrointestinal system endoscopy.

The severity of skin involvement was assessed using the modified Rodnan skin score (mRSS). This score evaluates the patient's skin thickness, based on a rating performed using clinical palpation, using a 0 - 3 scale for 17 anatomic areas of the body. The skin thickness is assessed by a scoring performed between 0 and 3, and defined as follows: (0 = normal, 1 = mild thickening, 2 = moderate thickening, and 3 = severe thickening). The total score is 51 [5]. The SSc disease severity was assessed by the utilization of the Medsger Severity Scale (MSS). In accordance with this scaling system, nine organ systems were scored. These organ systems were as follows: general health, peripheral vascular, skin, joint and tendon, muscle, gastrointestinal, lung, cardiac, and renal. Each organ system was scored between 0 (no involvement) and 4 (severe involvement) [6].

All thyroid ultrasound (US) examinations were performed by an experienced radiologist on the same US equipment. The radiologist had no information about the patients. During the US examinations, the thyroid gland was scanned both longitudinally and transversally for precise gland dimension measurements and nodule detections. All US examinations were performed on an Esaote Mylab 70 US (Esaote Group, Genoa, Italy) scanner by the utilization of a 7.5 MHz linear transducer.

The volumes of the thyroid glands were calculated according to the ellipsoid formula: volume (mL) = depth (cm) × width (cm) × length (cm) × π/6.

Thyroid nodules were defined as lesions of a diameter of at least 1 cm, located in and distorting the uniform shape or echo pattern of the thyroid gland. The volumes of the nodules were not considered within and as a component of the thyroid gland volume. According to the literature, the mean thyroid volumes for our region, women, and men are reported to be 12.09 ± 2.05 ml and 14.53 ± 2.55 ml, respectively [7]. Glands with an estimated volüme <4.5 ml (females) and < 5.5 ml (males) were considered atrophic [8,9].

Antinuclear antibody (ANA), anti-topoisomerase 1 (anti-scl-70), anti-centromere antibody (ACA) were recorded from the hospital database. Anti-thyroglobulin (anti-TG) and antithyroperoxidase antibodies (anti-TPO), hemogram, and biochemical tests performed in the last six months were also recorded from the hospital database. Venous blood samples for thyroid hormones were obtained the morning of the US examination, following overnight fasting. The blood samples of patients receiving intravenous (iv) prostaglandin therapy were obtained before the prostaglandin infusion. Levels of free thyroxine (T4), free triiodothyronine (T3), and thyroid-stimulating hormone (TSH) were measured and recorded. They were analyzed using the electrochemiluminescence ımmunoassay method and Roche Elecsys E170 device (Roche Diagnostics GmbH, Mannheim, Germany). A TSH level above 5.6 mIU/L, in association with decreased levels of free T3 and T4, was accepted as a sign of primary hypothyroidism, whereas a TSH level below 0.24 mIU/mL, in association with increased levels of free T3 and T4, was accepted as a sign of primary hyperthyroidism.

The Ethics Committee approved the study protocol of the University of Cukurova (approval date: July 15, 2019; approval number: 90). Written informed consent was obtained from all patients in accordance with the Declaration of Helsinki.

Statistical analysis

All analyses were performed by the utilization of the IBM Statistical Package for Social Sciences (SPSS) version 20.0 (IBM Corp., Armonk, NY, USA). Categorical variables were expressed as numbers and percentages, whereas continuous variables were summarized as the mean, median, minimum - maximum, standard deviation, and values. For comparing the continuous variables between the two groups, the Student's t-test or the Mann - Whitney U test was used, depending on whether the statistical hypotheses were fulfilled or not. On the other hand, the Pearson Correlation Coefficient or the Spearman Rank Correlation Coefficient method was utilized to evaluate the correlations between the measured values.

Logistic regression analysis was performed to determine significant predictors of thyroid volume variable. In univariate analysis, variables significant at the P <0.1 level were entered in logistic regression analysis. Adjusted for age and gender variables (adjusted logistic regression). The statistical level of significance for all tests was considered to be 0.05.

## Results

Patient features

The mean age of the patients was calculated as 52.7 ± 10.1 years. The male/female ratio was found to be 7/79 (9.3%/90.7%). There were 30 (34.9%) patients with limited SSc subtype, while 56 (65.1%) patients had the disease's diffuse subtype. Demographic, clinical, and biochemical features of the study cohort are summarized in Table 1.

**Table 1:** Clinical characteristics of patients ILD: Interstitial Lung Disease, PAH: Pulmonary Arterial Hypertension, mRSS: Modified Rodnan Skin Score, MSS: Medsger Severity Scale, ANA: Antinuclear Antibody, SCL–70 Ab: Anti–Topoisomerase I, ACA: Anti-Centromere Antibody

Age (years), mean ± std	53.5 ± 10,1
Female sex, n (%)	78 (90.7)
Diffuse type, n (%)	56 (65.1)
Limited type, n (%)	30 (34.9)
Disease duration (months) median (min-max)	96 (5- 480)
Immunosuppressive, No, n (%)	46 (53.5)
Mycophenolate mofetil	4 (4.7)
Cyclophosphamide	36 (41.9)
Smoked ever, n (%)	8 (9.3)
Presence of ILD, n (%)	52 (60.5)
PAH, n (%)	17 (%19.8)
Gastrointestinal involvement, n (%)	58 (67.4)
mRSS, mean ± SD	23.13 ± 12.78
MSS, mean ± SD	7.66 ± 4.76

Thyroid volume findings

Thyroid volume was in the atrophic range in 46 (53.5%) of the patients. In two (2.3%) patients, the thyroid volume was found to have increased. Together with those with normal thyroid volumes, these patients were evaluated as "patients whose thyroid volumes had not decreased." Statistically significant negative correlations were found between the thyroid volume and mRSS (p <0.001, r = 0.871), MSS (p <0.001, r = -0.563), and disease duration (p <0.001, r = -0.705) (Tables 2, 3).

**Table 2:** Associations among the thyroid volume, mRSS, and MSS mRSS: Modified Rodnan Skin Score, MSS: Medsger Severity Scale, TSH: Thyroid-Stimulating Hormone

Thyroid Volume	Non- decreased	Atrophy	p-value
Age (years), mean ± std	48.98 ± 8.8 50	56.02 ± 10.23	0.001
Duration of SSc (months), median (min-max)	48 (5- 300)	256 (24- 480)	< 0.001
MSS, median (min-max)	4 (2 -13)	10 (3- 24)	< 0.001
mRSS, median (min-max)	10 (2- 36)	34 (1- 43)	< 0.001
TSH (mlU/L), median (min-max)	5 (2- 11)	3 (0.1 – 5.2)	<0,001

**Table 3:** Correlations between the variables mRSS: Modified Rodnan Skin Score, MSS: Medsger Severity Scale, TSH: Thyroid-Stimulating Hormone

	Thyroid volume	Duration of SSc	MSS	mRSS	TSH
Thyroid volume	1				
Duration of SSc	- 0.731	1			
MSS	- 0.611	0.674	1		
mRSS	- 0.857	0.718	0.694	1	
TSH (mlU/L)	- 0.701	0.613	0.581	0.743	1

The mean thyroid volume of patients who had the diffuse type of SSc (6.365 ± 4.9 ml) was significantly lower than that of those who did not (p = 0.015). As can be seen from the table, the mean thyroid volume of the patients who had an interstitial lung disease was significantly lower than that of those who did not (p <0.001) (Table 4). No statistically significant relation was found between patients with a history of immunosuppressive agent consumption and those without (p = 0.06).

**Table 4:** Associations between thyroid volume levels and patient characteristics ILD: Interstitial Lung Disease, PAH: Pulmonary Arterial Hypertension, ANA: Antinuclear Antibody, SCL–70 Ab: Anti–Topoisomerase I, ACA: Anti-Centromere Antibody, SSc: Systemic Sclerosis

Thyroid volume		Normal	Decreased	Total	
		n (%)	n (%)	n (%)	p
Sex	F	36 (46.1)	42 (53.8)	78 (90.7)	>0.05
	M	4 (50)	4 (50)	8 (9.3)	
Immunosuppressive	Negative	25 (55.3)	21 (44.7)	46 (53.5)	>0.05
	Positive	15 (36.6)	25 (63.4)	40 (46.5)	
Smoking	Negative	36 (46.2)	42 (53.8)	78 (90.7)	>0.05
	Positive	4 (50.0)	4 (50.0)	8 (9.3)	
Raynaud	Negative	2 (66.7)	1 (33.3)	3 (3.5)	<0.001
	Positive	38 (45.9)	45 (54.1)	83 (96.5)	
ILD	Negative	25 (74.3)	9 (25.7)	34 (39.5)	<0.001
	Positive	15 (28.3)	37 (71.7)	52 (60.5)	
PAH	Negative	36 (52.1)	33 (47.9)	69 (80.2)	<0.001
	Positive	4 (23.5)	13 (76.5)	17 (19.8)	
Gastrointestinal involvement	Negative	18 (66.7)	10 (33.3)	28 (32.6)	0.007
	Positive	20 (36.2)	36 (63.8)	58 (67.4)	
Thyroid autoantibody	Negative	32 (43.4)	42 (56.6)	74 (86.4)	>0.05
	Positive	8 (66.7)	4 (33.3)	12 (13.6)	
ANA	Negative	2 (66.7)	1 (33.3)	3 (3.5)	>0.05
	Positive	38 (45.9)	45 (54.1)	83 (96.5)	
SCL 70 Ab	Negative	20 (55.6)	16 (44.4)	36 (41.9)	>0.05
	Positive	20 (40.4)	30 (59.6)	50 (58.1)	
ACA	Negative	25 (40.6)	37 (59.4)	62 (72.1)	>0.05
	Positive	15 (62.5)	9 (37.5)	24 (27.9)	
SSc subtype	Limited	20 (64.5)	10 (35.5)	30 (34.9)	0.013
	Diffuse	20 (36.8)	36 (63.2)	56 (65.1)	

Thyroid hormones and autoantibodies

The mean TSH value for the entire group of SSc patients was 4.39 mIU/L (min. 0.1; max. 11.1; median 4.2), whereas the prevalence of thyroid antibody positivity was found to be 12 (13.9%). Forty-six (53.5%) of our patients were euthyroid. Subclinical hypothyroidism was present in 21 (24.4%) patients, while 18 (20.9%) patients demonstrated hypothyroidism, and one (1.2%) patient demonstrated hyperthyroidism findings. A statistically significant negative correlation was found between the thyroid volume and TSH levels (p <0.001, r = 0.701). No statistically significant relation was found between the thyroid volume and thyroid autoantibody positivity (p >0.05). A positive correlation was found between the TSH levels and the MSS score (p <0.001, r = 0.573). Still, another positive correlation was present between the TSH levels and the mRSS values (p <0.001, r = 0.706).

Other findings

A solitary thyroid nodule was present in 11 (12.8%) patients, whereas 36 (41.9%) patients presented with multiple nodules. All nodules were detected incidentally.

According to the logistic regression analysis, an increase of 1 unit in the mRSS score increases the thyroid volume reduction risk by 1.377 fold [95% CI (1.126-1.683)]. And an increase of 1 unit in the disease duration score increases the thyroid volume reduction risk by 1.023 fold [95% CI (1.003-1.043)] (Table 5).

**Table 5:** Forward univariate logistic regression of the significant variables in groups with and without increased thyroid volume values mRSS: Modified Rodnan Skin Score, MSS: Medsger Severity Scale

Variable	Category	Coefficient	Std error	Wald	P value	OR (95%CL)
Sex	Male	Reference				
	Female	1,177	2,238	0,277	0,599	3,246(0,040-260,595)
mRSS		0,320	0,103	9,709	0,002	1,377(1,126-1,683)
Disease duration		0,023	0,01	5,316	0,021	1,023(1,003-1,043)
MSS		0,051	0,176	0,084	0,771	1,052(0,746-1,485)
Age		-0,021	0,056	0,143	0,706	0,979(0,877-1,093)
Immunosuppressive	No	Referance				
	Yes	-6,913	1,887	3,718	0,054	0,023(0-1,064)

## Discussion

Numerous studies exist in the literature pointing to the increase in the prevalence of thyroid dysfunction in SSc patients. But, up to our knowledge, there are no studies focused on the relations between SSc disease severity and thyroid volume alterations. Our study is the first of its kind to investigate the correlations between the thyroid volume changes in SSc patients and the mRSS and MSS. Our study demonstrated that in more than half of our patients the thyroid volume was at the atrophic range. The study also disclosed a negative correlation between thyroid volume and mRSS and MSS levels, and also disease duration.

Yao et al. [10] have performed a meta-analysis of SSc papers and reported a high prevalence of thyroid dysfunction in these patients. The authors have also emphasized a statistically significant relation between SSc and increased thyroid dysfunction risks. Borderline high levels of TSH (even if within the normal limits of range), together with TPO autoantibody positivity, presence of hypoechoic patterns, and presence of a small thyroid gland, have all been defined as risk factors for the development of thyroid dysfunction [11].

Literature data indicate a thyroid autoantibody positivity in SSc patients, in contrast to the controls. But it is reckoned that the biggest contribution to thyroid dysfunction comes from thyroid fibrosis [12].

Antonelli et al. [12] have reported from their study that they had found that hypothyroidism seen in SSc was correlated with thyroideal hypoechoic patterns and small thyroid volumes. We, too, found out at the end of our study, that there was a statistically significant negative correlation between thyroid volume and TSH levels (p <0.001, r = -0.701). We think that the correlation between TSH levels and disease duration and severity may implicate a relation between thyroid volume and thyroid dysfunction.

D'Angelo et al. [13], on the other hand, have reported the presence of fibrosis in 24% of the patients and 7% of the controls in an autopsy series comprised of 58 cases. A study performed by Singh et al. [8] on a group of 106 SSc patients has demonstrated that the thyroid gland volume was under 4.5 ml in 57.5% of the patients. No statistically significant correlations were found between the thyroid gland volumes and the TSH, anti-TPO antibody, anti-thyroglobulin antibody, levels (p = 0.076, p = 0.377, and p = 0.378, in respective order). In our study, the thyroid gland volume correlated with the extent of the disease. Statistically significant negative correlations were found between the thyroid volumes and the mRSS, MSS, and disease duration, values (in respective order: (p <0.001, r = -0.871), (p <0.001, r = -0.563), and (p <0.001, r = -0.705). No correlation was found between the thyroid gland volumes and thyroid autoantibody positivity (p >0.01). We found that the thyroid gland volume is lower in patients with a longer duration and higher severity. We think that this is related to the abundance of fibrosis in the gland.

Fibrosis of the thyroid gland can cause subclinical hypothyroidism or lead to conspicuous hypothyroidism [12]. Hypothyroidism may lead to clinical situations such as fatigue syndrome, hair loss, cold intolerance, constipation, skin changes, and musculoskeletal symptoms. These clinical situations, in turn, may further aggravate the symptoms of SSc [14]. This is why an early diagnosis and treatment of thyroid dysfunction is crucial.

Singh et al. [8] have reported that the prevalence of subclinical and apparent hypothyroidism is high (8.5% and 1.9%, respectively). The authors have also reported that a small thyroid volume (<4.5 ml) is associated with hypothyroidism. Another study has reported the presence of hypothyroidism in 16, and subclinical hypothyroidism in 14, of the 79 patients which comprised the study cohort [15]. Forty-six (53.5%) of our patients were euthyroid. Subclinical hypothyroidism was found in 21 (24.4%) of our patients, while 18 (20.9%) patients had overt, and one (1.2%) had subclinical hyperthyroidism. A statistically significant negative correlation was found between the TSH and thyroid volume levels (p <0.001, r = -0.701). On the other hand, a positive correlation was found between the TSH levels and the MSS and mRSS (p <0.001). A study performed by Antonelli et al. [12] has confirmed the findings of previous studies that demonstrated that thyroid dysfunction is more associated with thyroid volüme than thyroid autoantibody presence. The fact that TSH levels are higher in SSc patients with higher disease activity and longer disease durations point to a priority of fibrotic changes in the development of this process.

It has been reported in the literature that there was a statistically significant relationship between the serum TSH levels and mRSS in SSc patients. This study also reported that TSH and its receptor, TSHR, had a crucial role in the development of fibrotic alterations of the skin. Another important data related to this subject is that reports indicate the presence of TSHR messenger RNA in skin biopsies, cultured primary keratinocytes, and, more interestingly, dermal fibroblasts [16]. This can explain the positive correlation we found between TSH levels and mRodnan scores at the end of our study (p <0.001, r = 0.706).

When patients with atrophic thyroideal volumes were considered in our study, it was seen that the mean thyroid volume was smaller in patients with pulmonary hypertension when compared to those without (4.935 ± 4.8 ml; p = 0.012). Marasini et al. [15] have reported from their study that pulmonary pressures of hypothyroidism SSc patients were found to be significantly higher than those of euthyroid ones (p <0.05). The fact that the thyroid autoantibody was of low frequency in these patients points more to the thyroid hormones' vasomotor effect than an autoimmune mechanism. Singh et al. [8] have reported a positive correlation between thyroid volume and forced expiratory volume (FEV1) and forced vital capacity (FVC). On the other hand, the authors have reported a negative correlation between TSH levels and FEV1 and FVC. In our study, too, the thyroideal volumes were found to be significantly lower in patients with interstitial lung disease, in comparison to those without (p <0.001). Our study found that pulmonary involvement was more substantial in patients with a lower thyroideal volume (p <0.001). It can be said that the common cause is fibrosis. Evaluation of thyroid functions and volume will be appropriate in patients with ILD.

Animal studies have shown that cyclophosphamide therapy leads to structural and metabolic alterations in the thyroid gland. Our study could not detect any differences in terms of thyroid gland volumes between patients with and without a history of cyclophosphamide administration (p = 0.05).

Another incidental ultrasonographic finding we detected in our patients was the presence of thyroideal nodules. We encountered solitary nodules in 11 (12.8%) and multiple nodules in 36 (41.9%) of our patients. The sonographic prevalence in our country has been found to be 23.5% for the 18- to 65-year-old age group, whereas it is reported to reach a much higher level of 37.5% in the over-65-year-old age group. There are cases reported in the literature pointing to a relation between SSc and thyroid adenoma or cancer [17-19]. Antonelli et al. [12] have shown in their study that the thyroid nodule and papillary cancer prevalences were higher in their group of 327 SSc patients in comparison to the control group. Considering the increased malignancy risk in SSc, we propose a malignancy rule-out in SSc patients with ultrasonographically detected incidental thyroid nodules.

Our study had its own limitations. These can be summarized as the presence of a rather small population of patients, the absence of controls, and the lacking of thyroid biopsies. The absence of a control group was the limitation of our study. We did not have a control group, but in a study conducted with a healthy population in our region, the mean thyroid volume for women and men was reported as 12.09 ± 2.05 ml and 14.53 ± 2.55 ml, respectively [7]. Besides, in the literature, in a study conducted with 107 rheumatoid arthritis patients (81 females, 26 males, 22-78 years, median age 56) and a healthy control group, the mean thyroid volume was found to be 20.9 and 16 ml, respectively. They found no relationship between rheumatoid arthritis activity and thyroid volume and function [20].

There are very few studies in the literature evaluating the thyroid volume and clinical and demographic features in SSc patients. This is the first study evaluating the relationship between disease activity scores and duration. In addition, most of the literature on the subject consisted of studies performed on patients with the limited subtype of the disease. In our study, on the other hand, we examined the results we obtained from patients with the common subtype of the disease.

## Conclusions

In this study, we evaluated the thyroid volume in our SSc patients. We found that in more than half of our patients the thyroid volume was at the atrophy range. We also found that this situation became more conspicuous in patients with longer disease duration and higher disease severity. We disclosed a negative correlation between thyroid volume and TSH levels. We also made the observation that thyroid atrophy was more frequent in patients with ILD. Thyroid hormone dysfunction which may develop due to thyroid atrophy may aggravate SSc symptoms and contribute to patients' morbidity and mortality by affecting many systems. Our findings strongly indicate that patients with a long duration of the disease and high disease severity scores and skin thickness should be followed up periodically for thyroid dysfunction, and thyroid dysfunction should be considered in symptoms such as dyspnea, fatigue, and edema that do not regress with ILD remission.
